# A new validated T2* analysis method with certainty estimates for cardiac and liver iron load determination

**DOI:** 10.1186/1532-429X-17-S1-P52

**Published:** 2015-02-03

**Authors:** Sebastian L Bidhult, Christos G Xanthis, Love Lindau Liljekvist, Gerald F Greil, Eike Nagel, Anthony H Aletras, Einar Heiberg, Erik Hedström

**Affiliations:** 1 Lund Cardiac MR group, Department of Clinical Physiology, Lund University, Lund, Sweden; 2 Department of Biomedical Engineering, Faculty of Engineering, Lund University, Lund, Sweden; 3 Division of Imaging Sciences and Biomedical Engineering, King's College London, London, UK; 4 BHF Centre of Research Excellence and NIHR Biomedical Research Centre, Guy's and St Thomas NHS Foundation Trusts and King's College London, London, UK; 5 Department of Diagnostic Radiology, Lund University, Lund, Sweden; 6 Computer Science and Biomedical Informatics, University of Thessaly, Lamia, Greece

## Background

Accurate quantification of iron load is of importance in tailoring the therapy for patients with iron load disease. Magnetic resonance (MR) imaging is commonly used to assess iron load in different organs by calculating the T2* value. Estimation of precision and uncertainty of the obtained T2* value may be useful for determining changes in iron load between follow-up scans and for titration of treatment. Therefore, the purpose of this study was to develop and validate a new T2* analysis method, providing certainty estimates for quantification of iron load in heart and liver.

## Methods

The proposed method is a region of interest (ROI)-based algorithm which changes curve-fitting procedure based on the results from an initial T2* estimate using a 3-parameter, noise-corrected model. The certainty estimate, presented as the T2* value confidence interval, is derived from multiple sub-regions within the selected ROI. Except for ROI delineation, the proposed method does not require any user interaction.

Phantom imaging was performed on a 1.5T system, with two clinically available multi-echo gradient-recalled echo sequences for cardiac and liver imaging. Phantoms were constructed to cover the clinically important range of T2*. A T2* single-echo gradient-echo sequence with TR set to 6 × T1 and a 50° flip angle was used as reference standard. Computer simulations were performed to assess accuracy and precision from 2 000 repetitions at SNR=15. Inter- and intra-observer variability was obtained in patients (n=22) by one experienced and one inexperienced observer.

## Results

**Figure 1:**
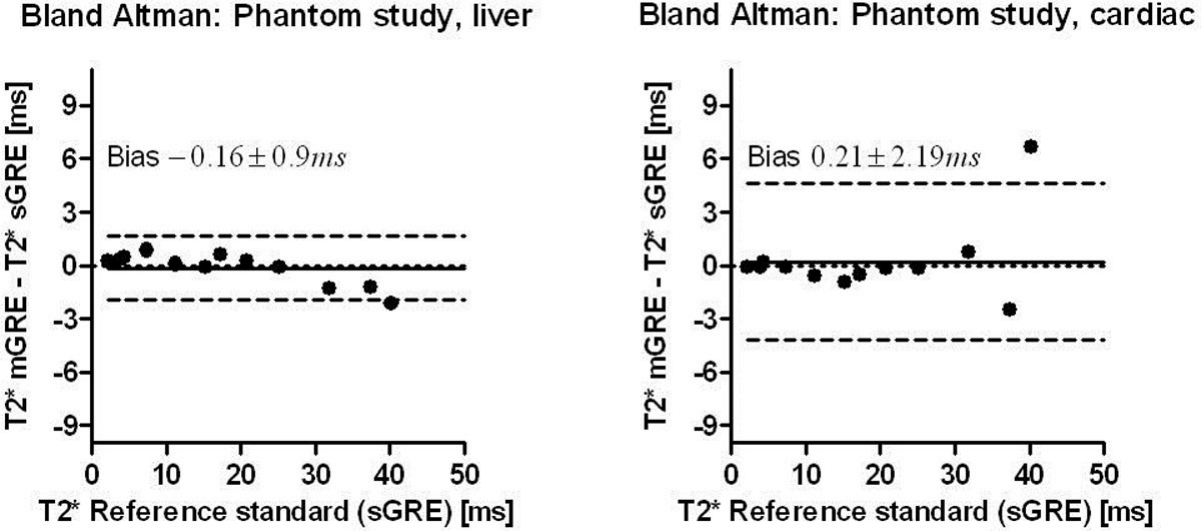
Bland-Altman analyses for the phantom study. The dashed lines indicate ±1.96 SD and the solid lines indicate bias.

**Figure 2:**
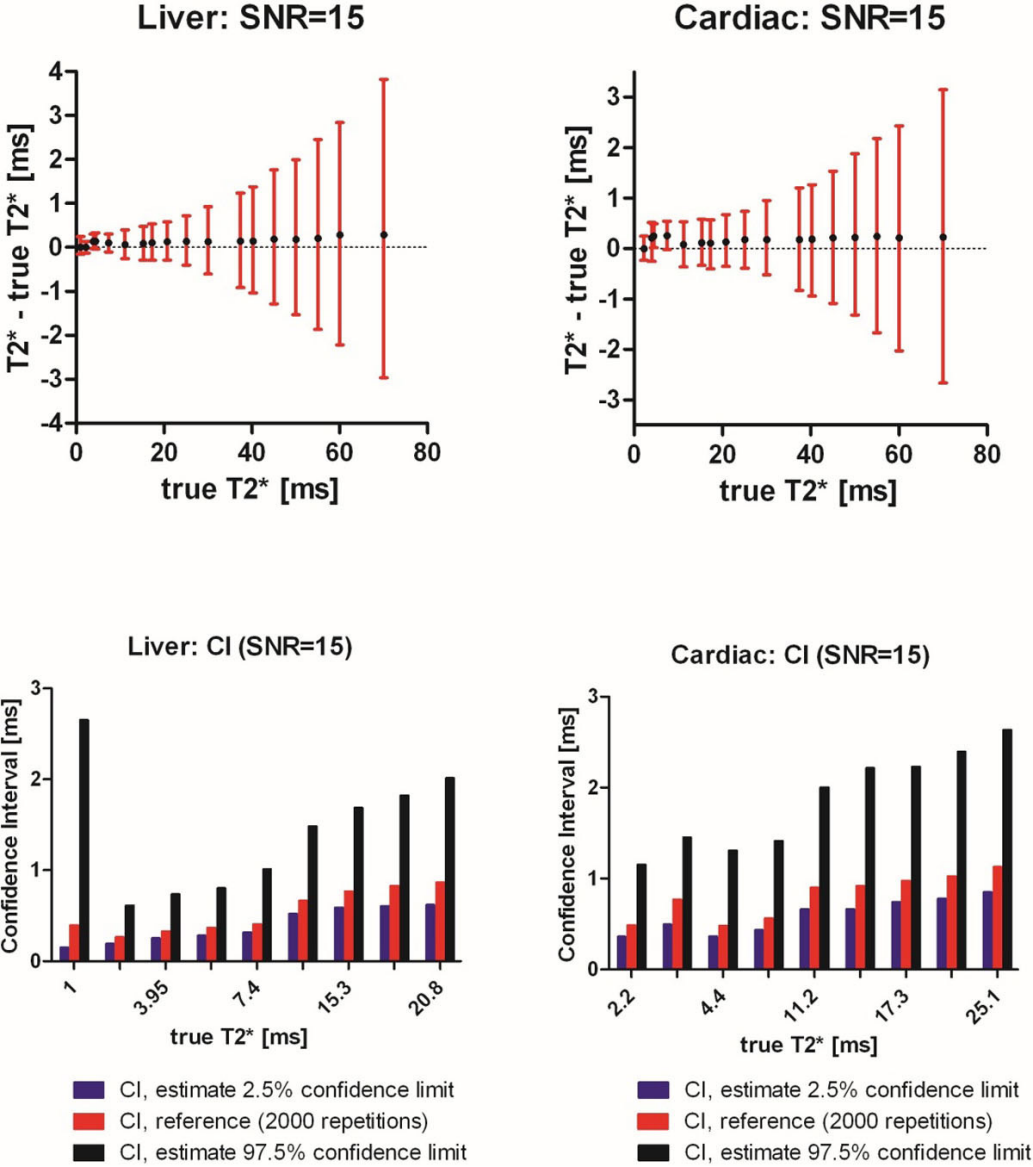
Computer simulations showing T2* accuracy, precision and certainty estimates. Top row: Differences in measured and true T2* using echo times from liver and cardiac sequences. Black dots indicate mean error of measured T2*. Red bars indicate confidence intervals (CI). Bottom row: Comparison of estimated CIs (blue and black) and the reference (red) from 2 000 repetitions, which lies within the confidence limits of the estimated CIs.

The phantom study showed bias and variability of 0.21 ± 2.19 ms (bias ± SD) and -0.16 ± 0.90 ms, for cardiac and liver sequences respectively (Figure 1). Simulation results are shown in Figure 2. The proposed method demonstrated high accuracy and precision, with certainty estimates close to confidence intervals derived from 2 000 repetitions. In patients, intra-observer and inter-observer variability was -0.03 ± 0.76 ms (bias ± SD) and 0.27 ± 1.89 ms, respectively.

## Conclusions

The proposed method accurately quantifies T2*, also providing T2* certainty estimates. The algorithm is freely available for research purposes.

## Funding

The Swedish Heart-Lung Foundation; the Medical Faculty of Lund University, Sweden; Skane University Hospital Lund, Sweden; the Swedish Societies of Medicine, Radiology and Cardiology, Sweden; Region Scania, Sweden; the Swedish Research Council; Wellcome Trust, UK; British Heart Foundation, UK.

